# An IoT-Based Gamified Approach for Reducing Occupants’ Energy Wastage in Public Buildings

**DOI:** 10.3390/s18020537

**Published:** 2018-02-10

**Authors:** Thanasis G. Papaioannou, Nikos Dimitriou, Kostas Vasilakis, Anthony Schoofs, Manolis Nikiforakis, Fabian Pursche, Nikolay Deliyski, Amr Taha, Dimosthenis Kotsopoulos, Cleopatra Bardaki, Sarantis Kotsilitis, Anastasia Garbi

**Affiliations:** 1 Department of Informatics, Athens University of Economics and Business (AUEB), 10434 Athens, Greece; 2 European Dynamics S.A., 1060 Brussels, Belgium; nikos.dimitriou@eurodyn.com (N.D.); kostas.vasilakis@eurodyn.com (K.V.); anastasia.garbi@eurodyn.com (A.G.); 3 Wattics Ltd., Dublin 8, Ireland; anthony.schoofs@wattics.com (A.S.); amr.druid@wattics.com (A.T.); 4 Plegma Labs S.A., 15125 Marousi, Greece; mn@pleg.ma (M.N.); sk@pleg.ma (S.K.); 5 Bosch Software Innovations GmbH, 88090 Immenstaad am Bodensee, Germany; fabian.pursche@bosch-si.com (F.P.); Nikolay.Deliyski@bosch-si.com (N.D.); 6 Department of Management Science & Technology, Athens University of Economics and Business (AUEB), 10434 Athens, Greece; dkotsopoulos@aueb.gr (D.K.); cleobar@aueb.gr (C.B.)

**Keywords:** energy efficiency, gamification, energy disaggregation, employee, behavioral economics, sustainability

## Abstract

Conserving energy amenable to the activities of occupants in public buildings is a particularly challenging objective that includes associating energy consumption to particular individuals and providing them with incentives to alter their behavior. This paper describes a gamification framework that aims to facilitate achieving greater energy conservation in public buildings. The framework leverages IoT-enabled low-cost devices, to improve energy disaggregation mechanisms that provide energy use and—consequently—wastage information at the device, area and end-user level. The identified wastages are concurrently targeted by a gamified application that motivates respective behavioral changes combining team competition, virtual rewards and life simulation. Our solution is being developed iteratively with the end-users’ engagement during the analysis, design, development and validation phases in public buildings located in three different countries: Luxembourg (Musée National d’Histoire et d’Art), Spain (EcoUrbanBuilding, Institut Català d’Energia headquarters, Barcelona) and Greece (General Secretariat of the Municipality of Athens).

## 1. Introduction

According to 2017 data, energy consumption in public buildings was 40% greater than that in residential buildings, while only 30% of non-residential buildings are public [1]. Apart from refurbishing and retrofitting older buildings to improve their energy efficiency, changing the consumption behavior of occupants in public buildings is considered a key factor that should be properly addressed, because “buildings don’t use energy, people do” [2]. This is a complex problem due to a lack of energy-consumption accountability as well as economic incentives (i.e., they do not pay the bill) for occupants in public buildings to conserve energy. Moreover, employees in these buildings are mainly pre-occupied with their everyday job tasks and are generally averse to any loss to their personal comfort at work.

In this paper, we describe a gamified framework that aims to change occupants’ energy-consumption behaviors and reduce energy wastage in public buildings. This framework has been developed as part of the CleAnweb Gamified Energy Disaggregation (ChArGED) project (http://www.charged-project.eu). By leveraging low-cost IoT devices (NFC/BLE), multi-channel smart meters and smart plugs, we improve prior energy disaggregation mechanisms [3] and identify energy wastages at the device, area and end-user level. At the same time, we employ a serious game approach, accessible through a mobile app, to engage and motivate users to save energy. This serious game involves team competition with intangible rewards and life simulation with the evolution of a virtual tree according to the performance of team members in energy-saving or energy-awareness challenges (i.e., watch a short educational video). An energy-saving challenge dictates an individual or team action for conserving energy and the accomplishment of any challenge awards some points to the respective actors.

Energy disaggregation in office buildings at the device level is particularly challenging due to physically large spaces, diverse energy appliance types, concurrent use of multiple similar appliances (e.g., PCs), several appliance instances and occupancy non-obstructiveness. Non-Intrusive Appliance Load Monitoring approaches (NIALM) have been investigated [4,5], but only for plug-level loads, however, the problem in general involves a trade-off between installing costly dense metering infrastructure and accuracy [6]. Our approach advances the state of the art in energy disaggregation in two ways: (i) all energy loads in the building are monitored and disaggregated at the device level in a cost-effective and highly accurate way; (ii) device usage is associated to specific individual(s) in the building; note that in public buildings multiple appliances are shared among multiple users and thus personalizing energy use is a key non-trivial contribution of our approach.

We have thoroughly studied and theoretically verified the potential effectiveness of our gamified approach–involving teaming, competition and rewards for energy conservation–through a detailed mathematical model in [7]. The actual effectiveness of our gamified framework to alter energy-wasting behaviours of occupants in public buildings will be evaluated through its deployment for a period of one year at the pilot sites of ChArGED in three different countries: Luxembourg (Musée National d’Histoire et d’Art), Spain (EcoUrbanBuilding, Institut Català d’Energia headquarters, Barcelona) and Greece (General Secretariat of the Municipality of Athens). The instrumentation of these pilot sites with the hardware of our framework is currently nearing completion.

There has also been some prior work on gamification approaches for conserving energy in public office buildings [8,9,10,11,12]. Cowley et al. [8] described a game framework aimed at conveying energy efficient knowledge and behaviors to users of public buildings by introducing “Behavlets” (i.e., action-resource-outcome triplets for educational purposes on energy savings). Different pilot sites belong to competing teams and the user will support a team by playing mini-games, taking quizzes, seeing ‘learn-more’ information pages about energy efficiency, and getting feedback in the form of real-time information about energy consumption in each one of the pilots. However, energy-saving actions themselves are not part of the game in [8], as opposed to our approach. Simon et al. proposed Climate Race [9], a pervasive game addressing office workers, where energy consumption is related to the activities of each player by observing corresponding behaviors through a comprehensive sensor and plug-level metering infrastructure, as opposed to our more cost-effective solution. Also, the GreenSoul approach [10] focuses on the reduction of the energy consumption in public buildings by means of a thorough sensor infrastructure that provides recommendations to occupants regarding energy savings. A virtual pet game designed for energy use reduction in a commercial office setting was introduced in [11], where device-specific energy consumption was reflected in the fitness of virtual pets. Our gamified app has far more ambitious goals than [11] in the sense that it aims to change a vast range of energy-wasting behaviors at work. In [12], a gamification approach with similar goals to ours is described, yet with a different game scenario and technological approach. They promote a list of actions that reduce energy wastage, and reward or penalize building occupants with redeemable virtual coins for following these actions. Peer pressure is exercised by means of observation of others’ actions and by clustering occupants with similar energy-consumption habits, while only belonging to the most energy-conserving cluster is rewarded; not belonging to this cluster is penalized. However, during our ethnographic studies and online surveys with the occupants of the pilot sites of ChArGED, we found that observing actions of others would violate their privacy concerns and that penalizing users for their non-energy efficient behavior would completely demotivate them to engage into the game in the first place. In the context of other public buildings, Amaxilatis et al. [13] aim to increase energy awareness and alter energy-consumption behaviors of children through educational activities in schools. Johnson et al. [14] reviewed multiple energy-saving competitions among university students and identified several pitfalls in their design. Specifically, the use of total energy consumption or (relative) energy-consumption reduction for winner determination is deemed as not adequate when static baseline calculation methods are employed and may be unfair for already “green” consumers. According to our approach, players obtain points by accomplishing game challenges for avoiding respective energy-wasting actions, which may be undertaken by players on a voluntary basis. Thus, “green” consumers compete against others from the same starting point. Furthermore, as (most of) the challenges are expected to be easily attainable by already “green” consumers, the latter have the advantage.

In addition, other related applications, e.g., Kill-Ur-Watts, Energy Tracker, Watts Plus, etc., mainly focus on increasing energy-consumption awareness, while assuming that the users are already interested in their energy consumption and motivated to reduce it. As the engagement of occupants in public buildings to such a game app cannot be taken for granted, we followed a carefully-designed gamified approach. Our approach employs both direct incentives and peer pressure to achieve the desired behavior change towards energy-consumption reduction, in terms of team competition with rewards.

In the general area of context-awareness for energy conservation in buildings, several approaches have been proposed [15,16,17]. These approaches employ sensory solutions (e.g., switch, luminosity sensors in [15], temperature and humidity sensors in [16], indoor localization solutions in [17]) to determine potential energy misuse depending on the actual device employed, the climate conditions and the user location.

The remainder of this paper is organized as follows: in Section 2, we overview the architecture of our system. In Section 3, we describe the heart of the data storage and communication of the system, namely the Data/Core Backend. In Section 4, we describe the basic principles of our game design based on the assessment of our users’ requirements. In Section 5, we present our IoT and energy metering infrastructure for observing behavioral changes of employees towards energy conservation and illustrate indicative energy consumption measurements for baseline creation. In Section 6, we describe our gateway solution for aggregating energy and sensor data. In Section 7, we overview our Energy Analytics Backend, where baseline calculation, energy savings and potential energy-saving opportunities are calculated. In Section 8, we present our Gamification System, i.e., the frontend and backend components of the game application. In Section 9, we complete the presentation of the system components by describing a solar-microgeneration forecasting system. In Section 10 and Section 11, we describe the operation of our system. Specifically, Section 10 overviews the call sequence for the implementation of three main processes in our system, while Section 11 describes the implementation of the validation of the accomplishment of one challenge. In Section 12, we describe our system evaluation methodology and some preliminary evaluation results. Finally, in Section 13, we conclude our paper and discuss future work.

## 2. System Architecture

**Figure 1:**
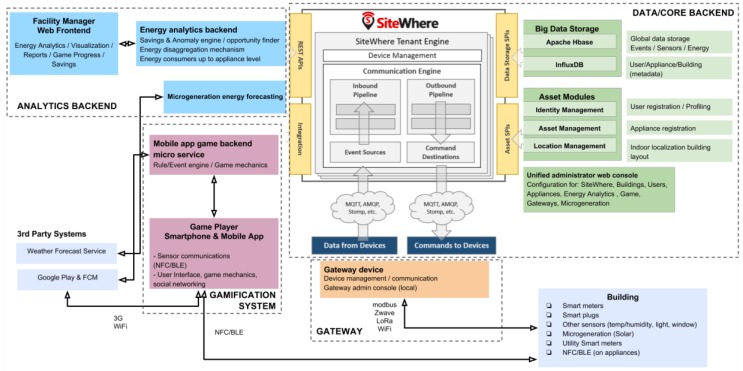
The ChArGED system architecture.

The logical architecture of our system (depicted in Figure 1) consists of four main groups of functional blocks: The Data/Core Backend group is responsible for providing an environment in which data, assets and users are stored and managed. The Backend components provide the software infrastructure on which the game app is developed. Even though that group of components is application agnostic, it is tuned towards the needs of the game. The Gateway group is responsible for integration of energy use and environmental data to the Backend system, in order to determine variations over the energy context within the building. The Energy Analytics Backend component is responsible for delivering insights that enable the gamified application to deliver custom and targeted feedback and incentives to the end-users. Finally, the Gamification System is responsible for processing field data and insights created from such data to make decisions as per the evolution of the game for each user, i.e., what the next step is towards additional energy savings. That group also delivers the mobile app, which the end-users interact with and which acts as an interface between the user and the rest of our system updating the user with the current game state and also provides information to the system about the users’ behavior towards the fulfillment of undertaken energy-saving challenges. The architecture also includes an external system that is utilized to provide a solar-power microgeneration forecast based on weather predictions and historical weather data for a specific location. It serves to help minimizing the daily energy load of the building by challenging the game players to time-shift inelastic energy-consuming actions towards periods where the net energy balance (i.e., microgeneration minus consumption) is maximized, when energy storage is not available.

## 3. Data/Core Backend System

The Data/Core Backend system components and infrastructure allow the overall platform to operate according to the requirements of the game challenges. SiteWhere [18] was chosen as the Data/Core Backend system for our application, providing an open-source platform with a number of rules and mechanisms for data exchange and operations. SiteWhere’s main functionality is to supply a server based JAVA SPRING middleware between the sensing infrastructure and the different system components and acts as a controller for the processing of device data. It connects with NoSQL & Time series databases in order to provide persistence of the sensor data and scales effectively with a large number of devices so that the whole sensor data history is maintained and can be accessed at any point. It also provides the entities management mechanism in order to structure the devices and categorize them according to their type, location and ownership and offers full control on a device’s lifecycle (providing the functionalities of creating, deleting, updating, grouping, sending data). Moreover it provides a web based administrative console application that allows all of the system data to be viewed and manipulated in a structured way which makes their overview and administration easier and more accessible. The available functionalities are the following:

- Each new asset or entity (i.e., a sensing device, an appliance, a specific location area, a person) is assigned a unique id and can be autonomously monitored via external software. Specifically, a model for standard types of generated event data is provided for each device (which includes measurements, alerts issued and location updated by the device). The logged events are stored in massively scalable time series datastores (InfluxDB).
- Devices (appliances such as printers, air conditioners, PCs etc.) can be assigned to/associated with other entities. A device can be associated with a person, a location or another sensor device of our infrastructure thus giving us the ability to establish ownership room/location metadata and establish relationships with device.
- Devices can be grouped together according to a common role they fulfil, thus enhancing efficiency by simplifying the way the devices can be retrieved by other backend processing services.
- Every top level entity is modelled as a tenant and can have a completely different configuration and structure without affecting other tenants. This can be used for modelling infrastructures that are unrelated to each other, e.g., different locations, buildings, pilot users, etc., at the same server.

SiteWhere provides an extensive list of third party frameworks and software tools that it can be connected with in order to extend its capabilities. The options include different databases, identity management frameworks, event streamers, event processors, enterprise service buses and others. Moreover being an open source software solution, new interfaces with other software tools and services can be created as needed. External communication with SiteWhere can be achieved via a built in extensive REST APIs. A communication interface utilizing the MQTT protocol is also implemented that can be used by devices and other embedded systems to send or receive notifications about new events, as well as sensor data (e.g., NFC/BLE alerts and energy measurements).

**Figure 2:**
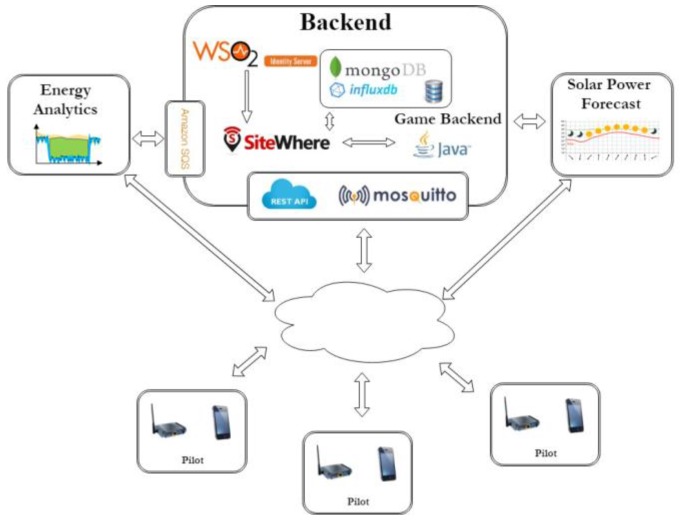
Organisation of SiteWhere system components in our solution.

The integration and connection of major system components is illustrated in Figure 2. The WSO2 Identity Server handles the identity management and user authentication, the Game Backend implements the logic to update the game progress of the users (i.e., score, completed challenges etc.) by processing the users’ actions, the Energy Analytics Backend performs estimations on user savings and is responsible for the energy monitoring of the building, and the mobile app acts as a frontend for the whole system as well as the main point of interaction with the users.

## 4. Game Design

Our gamified solution has been designed in an iterative Rapid User-Centered Design (Rapid UCD) process involving end-users for its analysis, design and development in public buildings located in three different countries: Luxembourg (Musée National d’Histoire et d’Art), Spain (EcoUrbanBuilding, Institut Català d’Energia headquarters, Barcelona) and Greece (General Secretariat of the Municipality of Athens).

To design an effective, appealing gamified application that the users will be willing to adopt, ethnographic-style studies (i.e., semi-structured interviews) and online surveys were conducted. The aim was to better understand the situation regarding the individual factors that influence energy consumption by the employees in the pilot sites, their current energy-consumption habits as well as in-game preferences, work engagement and burnout levels. In terms of individual behavioral factors, the following results summarize the majority of the users’ characteristics. The studies’ participants (potential pilot users) have been found to:

- Exhibit positive environmental awareness and environmental worldviews.
- Exhibit activated environmental personal norms, acknowledging that conserving energy and resources is important to them and their own problem.
- Show sensitivity to social norms, claiming that saving energy is a collective effort (doing it alone does not have an impact).
- Be willing to help their organization and to change their daily routine to conserve energy.
- Consider their personal comfort at work of crucial importance.

Thus, in general, employees from the pilot sites are positively positioned towards pro-energy conserving behavioral changes, as long as their personal comfort is not significantly affected. A more detailed account of the findings from the interviews we performed can be found in [19,20].

In terms of in-game preferences, most of the employees considered that only team efforts would be effective towards energy conservation at work. Also, a social competition would be of interest for the majority of employees, as long as individual performance was not exposed. Finally, employees find some sort of reward (monetary or not) motivating for energy conservation. These results have been considered for the implementation of the game mechanics and app.

In addition to the insight gathered from the semi-structured interviews and on-line survey, baseline data have also been collected and analyzed, to record the situation before the game is provided. The energy consumption data collection was implemented for all pilot sites with the targeted use of IoT smartplugs and N-channel meters before the deployment of the game. This resulted in the identification of interesting general energy wastage patterns which have been targeted by specific game challenges in our game (which have also been observed during the on-site visits and reported by users in the online surveys):

- Monitors are mostly left on stand-by when employees leave the office, even after switching-off their PCs.
- PCs may be left on after working hours.
- Printers are mostly left on after working hours.
- A/Cs are left on even when no employees are present at their respective spaces (in one building A/Cs are turned-off manually, while in the other two they are centrally controlled).
- Employees open windows whilst the A/C is on.

In addition, based on the data collected through on-site visits, as well as insight from the employee interview process, specific energy wastages have been identified for each individual site. This insight has enabled us to explore the exact usage patterns and electrical installation characteristics, in order to more accurately specify the game play conditions and the monitoring and game setup requirements at each site. Furthermore, the topology of each pilot site has been studied, to identify locations of teams and all electrical appliances, electrical circuits and controllers. Moreover, the various building spaces’ usage patterns have been studied, to specify what aspects should be monitored during the game execution such as, for example, to identify which devices are shared.

Based on the above, we defined the game around *energy-saving challenges*, each targeting a different existing energy-wasting behavior, e.g., do not open any windows, while A/C is on. Each challenge is defined in the Game Backend as a specific rule, which is validated based on sensor and energy-measurement data. A player may undertake a challenge and upon accomplishment is awarded a number of points and a challenge-specific badge. Building occupants belong to different teams and individual points contribute to team score, while teams compete against each other in a team competition setting. Building occupants may also undertake team challenges, the accomplishment of which is verified against all team members and awards points to all of them. An analytical evaluation of this team competition game setup with a detailed mathematical model based on behavioral data from the pilot sites of ChArGED in [7] has showcased the potential effectiveness of the approach to motivate building occupants to conserve energy. The winner team of a weekly/monthly team competition is awarded a corresponding virtual cup. As a player earns more points, its personal-accomplishment rank gradually changes from beginner to normal, then to pro and ultimately to expert. Also, within a team, members can earn the status of a captain or a deputy according to their respective individual performance, as compared to other team members. Finally, the progress of a player (resp. team) is visualized in the growth and the vitality of a personal (resp. team) virtual tree on a life-simulation basis.

## 5. Energy Metering and IoT Infrastructure

Commercial and industrial buildings are usually supplied with three-phase power to be able to deliver power to both single-phase end-loads, e.g., lighting and appliances, and three-phase end-loads, e.g., machinery and A/C. It is general practice to install power meters on the circuits of interest to capture information on their electrical energy use. Three-phase meters are generally used in non-residential settings to measure electrical energy consumption and relevant line parameters in all wiring configurations. Meters usually provide pulse or Modbus output and require data to be retrieved by a gateway or data acquisition system. Modern devices are now able to automatically send readings to a central database using push protocols such as HTTP, once provided with internet access, or other communication options typical of automated meter reading (AMR). Multi-channel three-phase meters, henceforth N-channel meters, are similar to N three-phase meters combined into one single device. N-channel meters are cost-efficient for monitoring multiple circuits fed from the same board or from multiple boards located in the same room. For example, with 12 channels, such a meter can combine a wide range of CTs with different amperage sizes, and can be used to monitor up to 36 × 1-phase circuits, 12 × 3-phase circuits or a mix of both. We employ N-channel smart meters (3 × 18-channel Accuenergy AcuREV Modbus meters at one site and 3 × 6-channel Accuenergy AcuRev 2020-2EM-NET-D 333mV meters at the other two sites (Accuenergy Canada Inc., Toronto, ON, Canada) to measure the load of multiple electrical circuits, e.g., room lighting, room fancoils, elevator, humidifier, room plugs, etc.

Office devices can be personal (e.g., PC, office lamp, etc.), or shared (e.g., printer, copier, coffee machine, etc.) among employees at the public building. We employ one plug-level meter per each shared device, while one channel of the N-channel meter or one plug-level meter is employed for up to four individual desks. Based on the device power signatures and common non-intrusive load monitoring techniques [3], the latter choice is known to enable accurate disaggregation of the load at the device level–given the few device categories that this small number of devices belongs to. Fibaro Z-Wave smart plugs have been used.

As we aim to change individual energy-wasting behaviours, energy consumption of each device needs to be associated to particular occupants at the public building. Bluetooth Low Energy (BLE) beaconing devices (Estimote Beacons, Estimote, Inc., San Francisco, CA, USA) are employed at office areas for proximity sensing. The distance of a smartphone of an occupant from a BLE beaconing device is estimated based on the received power and this information is forwarded to the Data/Core Backend System. Thus, when placed appropriately, BLE beaconing devices can detect presence of individuals at a specific room or an area, provided that these individuals carry their smartphones with them. Moreover, an NFC sticker is associated to each device (and lighting switch). The building occupants are requested to report their energy-saving actions to the system during the game, by swiping their phone over the various NFC stickers, in order to get awarded a number of points for the energy conserved. We have to emphasize that NFC swipes are mere claims of energy-saving actions on behalf of individual users. The Energy Analytics Backend validates the claim against electricity load measurement data and informs the Gamification System through the Data/Core Backend. Only then the user that performed the energy-saving action gets rewarded with points. This approach is *incentive compatible* for users to comply with. No NFC swipes are required by the users to report their use of a particular device. A Fibaro 4-in-1 Sensor (Temperature, Humidity, Luminosity, Motion/Presence) (Fibar Group S.A., Poznań, Poland) is also employed per area, in order to aid in the association of energy saving actions to particular individuals and in the discovery of energy-saving opportunities, as well as to monitor the comfort conditions in the building.

Overall, instead of following a comprehensive instrumentation approach where each individual energy load is individually monitored and all user activity in the workspace is constantly tracked, we follow a more cost-effective and less intrusive instrumentation approach, as described above, which is obviously less costly. Only energy-saving actions are monitored against well-known energy-wasting behaviors per deployment site. The actual energy saved per action is estimated according to the approach described in Section 7. The effectiveness of the approach will be measured during the pilot experiments of ChArGED.

**Figure 3:**
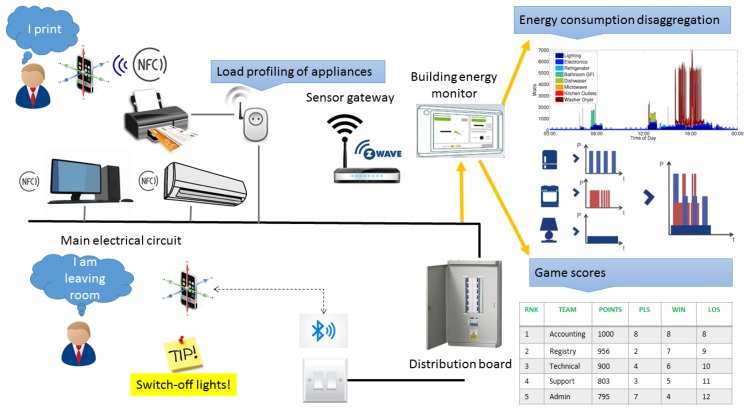
IoT and energy metering infrastructure for personalized load disaggregation.

Our instrumentation approach is summarized in Figure 3. All energy measurement and sensor data are forwarded to the Data/Core Backend System and employed to verify the accomplishment of game challenges for reducing energy wastage.

**Figure 4:**
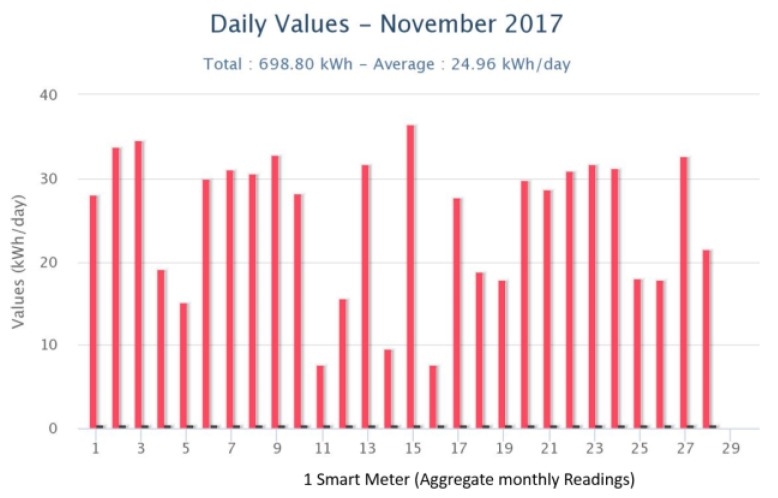
Energy measurements from smart meters in DAEM.

Next, we illustrate measurement data collected by the Accuenergy smart meters that have been installed in one of the pilot sites (DAEM). They show both aggregate measurements (representing all circuits belonging to one power meter) and individual circuit-level itemized data that represent a few or just even one appliance. The plots represent daily, weekly and monthly measurements and can be used an input to the Energy Analytics backend for creating energy-consumption baselines, as well as for identifying energy drops, anomalies and saving opportunities. Figure 4 depicts overall daily energy consumption at DAEM premises for a month period. As expected, energy load is much higher during weekdays than in weekends, except for occasional weekday exceptions (e.g., 14–16 November due to strikes in public transportation). 

**Figure 5:**
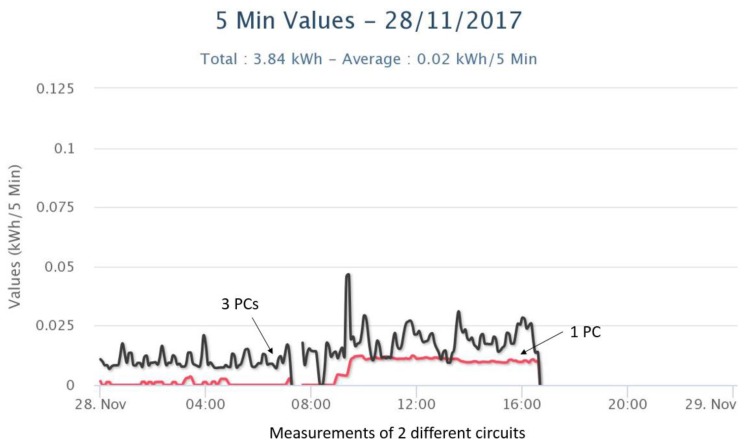
Energy measurements from smart meters (2 different circuits) in DAEM.

**Figure 6:**
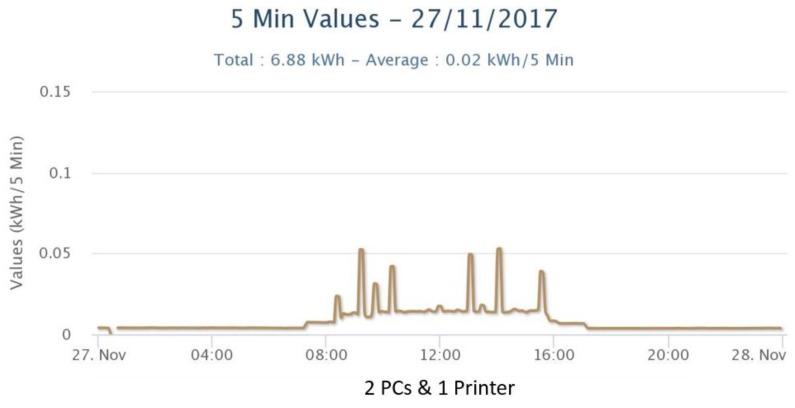
Energy measurements from smart meters (1 circuit) in DAEM.

Figure 5 and Figure 6 show energy measurements in 5 min time intervals from different electrical circuits at DAEM corresponding to different sets of devices. As shown in Figure 5 for time period before 8 a.m., some individual PCs are left on overnight. Also, spikes in Figure 6 correspond to printing events.

**Figure 7:**
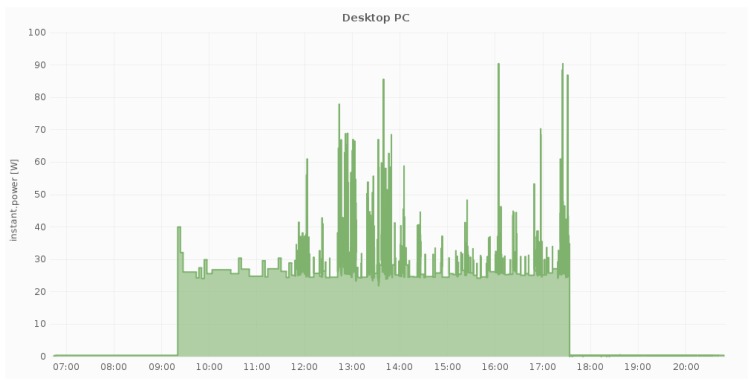
Power measurements from one smartplug at a personal device (1 PC).

**Figure 8:**
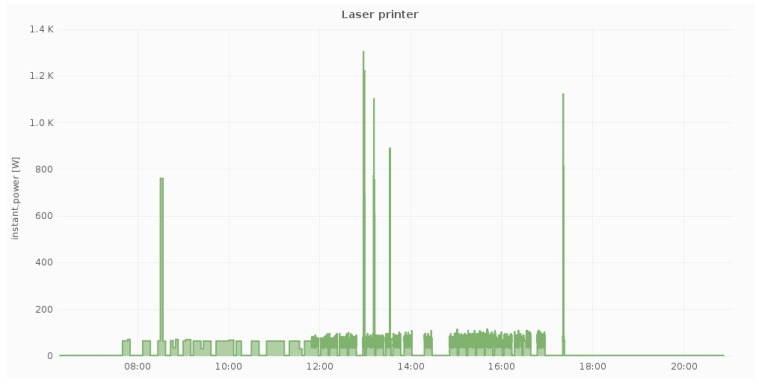
Power measurements from one smart plug at a shared device (1 laser printer).

**Figure 9:**
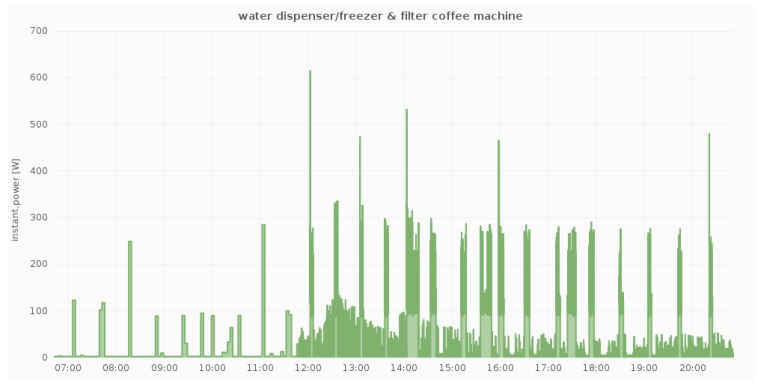
Power measurements from one smart plug (1 water dispenser/filter coffee machine).

Additionally, Figure 7, Figure 8 and Figure 9 illustrate the daily power measurements collected from the Fibaro smart plugs that have been installed in three different locations at a pilot site (DAEM). Load spikes in Figure 7 correspond to printing events, while, in Figure 8, they correspond to combination of the cooling cycles of the water dispenser and the use of the filter coffee machine. Overall, we measure electricity load in all circuits and for all devices whose load can be affected by user activity.

## 6. Gateway

To achieve the data acquisition process the Sensor Gateway has two connection interfaces within the global architecture:

- Building sensors (e.g., smart plugs and smart meters)
- SiteWhere Data/Core backend

Various hardware and software requirements have to be fulfilled to support the needs of the platform. The Sensor Gateway software/middleware by Bosch Software Innovations (Bosch Software Innovations GmbH, Immenstaad am Bodensee, Germany) is used as the basis of the Sensor Gateway in our system architecture. For the remote software management and provisioning of the product, the ProSyst Remote Manager (PRM) by Bosch Software Innovations [21] is also used. The Raspberry Pi (version 3 Model B) was chosen as the hardware basis for our Sensor Gateway. It is installed with a standard Raspian OS, including the Oracle Java Runtime Environment (Java8), the Communications Device Class Abstract Control Model (CDC_ACM) USB to serial driver and, as mentioned before, the ProSyst mBS SH Runtime for ChArGED.

**Figure 10:**
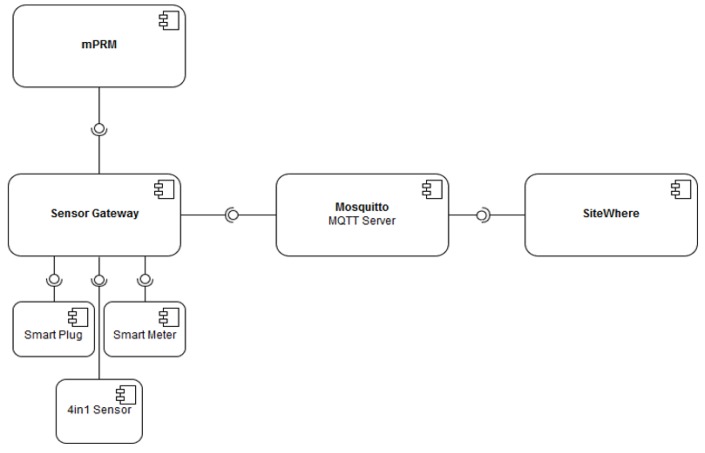
Illustration of the sensor gateway data collection, processing and communication to Sitewhere.

The data collection process (illustrated in Figure 10) required development of sensor drivers to retrieve data from third party sensors using industry leading communication protocols. For the connection of Z-Wave (Plus) devices, various controller options have been investigated, and two units have been selected: (1) “Razberry” GPIO Module for Raspberry Pi, (2) USB Z-Wave Controller.

Various Z-Wave devices were connected to the Sensor Gateway such as: (1) Fibaro Smart Plugs, (2) Fibaro 4-in-1 Sensor (Temperature, Humidity, Luminosity, Motion/Presence), (3) Fibaro Contact sensors.

These devices are managed by the mBS SH Runtime and included into the product portfolio, which allowed data to be immediately collected from the devices. All ZWave end devices that have metering functionality are registered automatically to SiteWhere. The Smart Plugs are configured to send events with their current power consumption or total energy consumption to the Sensor Gateway, when their measured values change. To reduce the transmission overhead to the backend, a configurable threshold is considered to filter out small value changes, ensuring that only significant changes are sent as new measurements to SiteWhere. The AcuRev 2000 multichannel Modbus meter by Accuenergy (Accuenergy Canada Inc., Toronto, ON, Canada)was also connected to the Sensor Gateway via the Modbus protocol, to collect detailed energy measurements at the three pilot sites. All connected devices communicate their data to the Sensor Gateway, which preprocesses and forwards it to the SiteWhere backend via MQTT. The default interval for the measurement polling of the Sensor Gateway to the Modbus devices is 20 s, but it can be reconfigured to reduce or increase the recorded data samples for the processing conducted in the Analytics Backend. For the management of the mBS Runtime the ProSyst Remote Manager (mPRM) backend is used. mPRM is a software and device management system developed by Bosch Software Innovations that enables lifecycle management of software bundles running in the mBS Runtime. Existing bundles can be updated, new bundles can be installed and deprecated bundles can be uninstalled. All of these actions are done during runtime; this means that all non-affected bundles are actively running, while the specific bundles are processed. Therefore, a 24/7 runtime of the sensor gateway is achieved.

## 7. Analytics Backend

**Figure 11:**
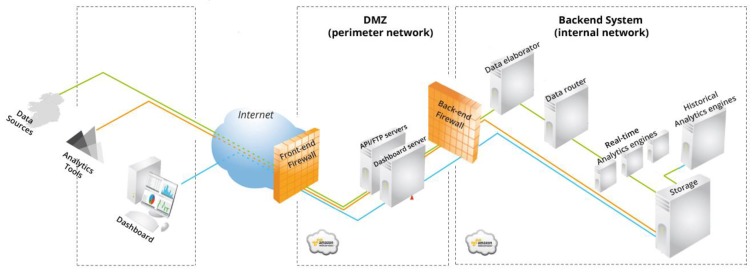
The Wattics cloud backend system architecture.

The Wattics Analytics Backend has to validate each action taken by a user related to energy savings, as the game relies on counting points based on the completion of challenges. Any such control action taken must lead to a reduction in energy use for a specific appliance (or group of appliances), which must be identified as the action occurs. As such the back-end analytics system must: (1) monitor energy use for a given appliance (or group of appliances) affected by a user’s control action by monitoring energy use within the premises at a granularity that allows for the energy use pattern of the specific appliances to be monitored individually, (2) detect changes in energy use over that circuit that relates to the NFC swipe from the user. The only commercially viable option, adopted within the project, is to submeter medium-level electrical circuits feeding groups of appliances (e.g., set of fancoil units in the same room), and run analytics on the data collected to monitor power variations of specific electrical equipment fed from such circuits. The Analytics Backend engine was developed to run on such medium-level circuit measurements in order to provide validation and estimation of savings achieved by a specific control action on an electrical appliance fed from that circuit. The validation that a control action claimed by a user has been really done is achieved by means of circuit-level measurements taken by physical meters, combined with metadata provided by the user NFC-swipe events and BLE beacon location sensors (and/or motion sensors). The principle of such analytics is energy-savings allocation, where energy reduction events are disaggregated from the power signal thanks to user-triggered notifications, and allocated to users associated to the equipment being operated. The Wattics Analytics backend is interfaced with the Sitewhere backend via RESTful web services, which allow energy measurements, NFC swipe alerts and BLE location events to be received as input, and measurements of load demand reduction, energy savings—as well as energy saving opportunities—to be returned. Authenticated data streams are processed in real-time through parallel analytics engines to produce valuable insights for the application. The Wattics backend infrastructure (illustrated in Figure 11) is brought to the project as background IP, and has been adapted to the needs of the project with the following additions:

- API endpoints to process NFC and BLE data packets.
- Analytics engine to validate control actions have been taken by users (e.g., device switched off when going home or when away for more than *N* minutes), and to estimate the energy savings achieved by such actions. In addition, the new Analytics engine is able to diagnose inefficient operation of electrical devices based on concurrent power activity (e.g., A/C left on when window is open), and to estimate the energy wasted due to such actions.
- Notification mechanism to export insights generated to Sitewhere. In addition to these extensions to the Wattics backend, a micro service was developed to reside in between SiteWhere and the Wattics Analytics backend to enable seamless integration of both backend systems via Amazon SQS.

**Figure 12:**
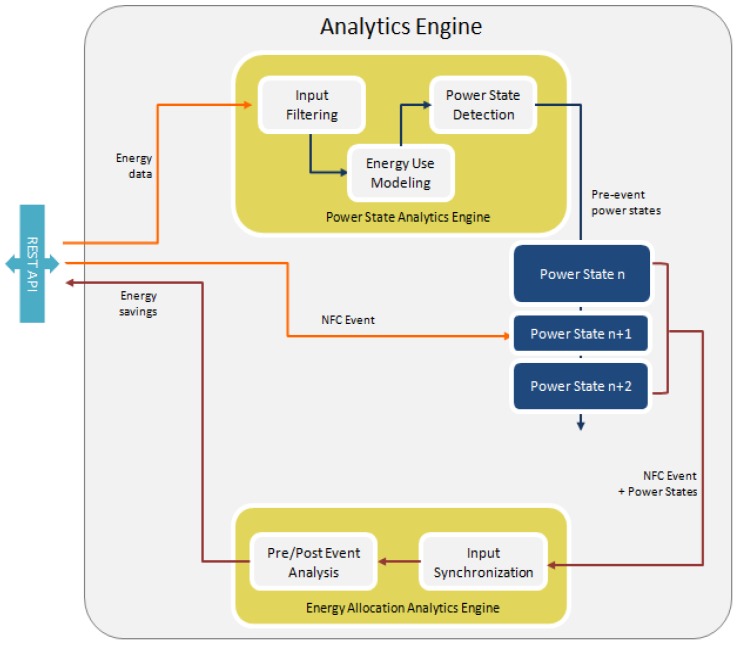
Architecture of the analytics engine in charge of validating user control actions and estimating energy saving generated.

The analytics component in charge of validating the control actions and estimating the savings generated has been implemented based on the architecture shown in Figure 12. The Pre/Post Event Analysis is where the algorithms for control validation and energy savings estimation happen. The disaggregation and energy allocation engine works as follows: (1) The core/backend platform informs the analytics engine that an appliance has been operated by the user after it received an NFC swipe alert from the user’s mobile app, (2) the analytics software runs the NFC swipe alert against the power measurements of the circuit feeding the appliance operated by the user to detect significant power variations, (3) the analytics software analyses the power variations and informs the core/backend platform of the drop in energy use measured in relation to the user’s operation of the appliance, as well as a quantification of the savings achieved by doing so and (4) the game backend calculates the points to be given to the user and the savings are stored within the platform database.

**Figure 13:**
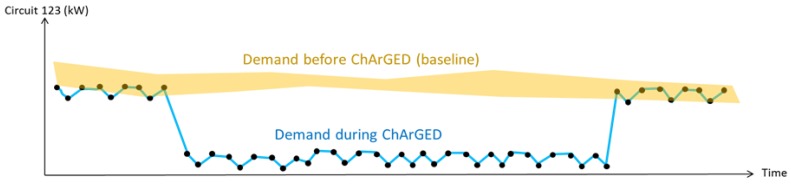
Load demand comparison under same conditions.

Calculating the savings on a specific circuit requires access to power measurements for that circuit before and during the operation of ChArGED, and measurement of external conditions that impact the energy used by equipment supplied from that circuit (e.g., work hours, weather conditions, etc.). With these data sets, it is possible to compare the energy use for a specific circuit when ChArGED system runs, against the baseline energy load for the same circuit observed in similar conditions without ChArGED. We aim to calculate savings achieved on individual circuits by multiple user actions, add them up over the trial period to have the total savings per circuit, and finally sum everything to have a total saving figure. Therefore, it all comes down to calculating individual savings on a circuit. The process consists of building for each circuit and each day of the week a model of the expected energy use before ChArGED, which can be compared against real measurements after ChArGED triggered control actions regarding energy-saving challenge accomplishment and associated NFC, BLE or motion sensor events. For the sake of accuracy, this model must depend on the day of the week and the external conditions that impact energy use, e.g., if the user makes an action on a lighting circuit in June on a Thursday at 17:00, then a model of similar conditions must be produced to compare to. This baseline model is produced by the proprietary Wattics Sentinel Analytics engine (a commercial product released in 2014 and already deployed for hundreds of customers) (Wattics Ltd., Dublin, Ireland) using data collected before the start of the trial. Sentinel uses a prediction algorithm that identifies days with similar conditions and creates a model of what the usual energy use typically is under such conditions before ChArGED was introduced. During the system operation, when a user triggers an action, the platform retrieves the correct baseline model based on current conditions and the new measurements are compared to the ones that would have been expected without ChArGED, which allows savings to be calculated, as illustrated in Figure 13.

## 8. Gamification System

**Figure 14:**
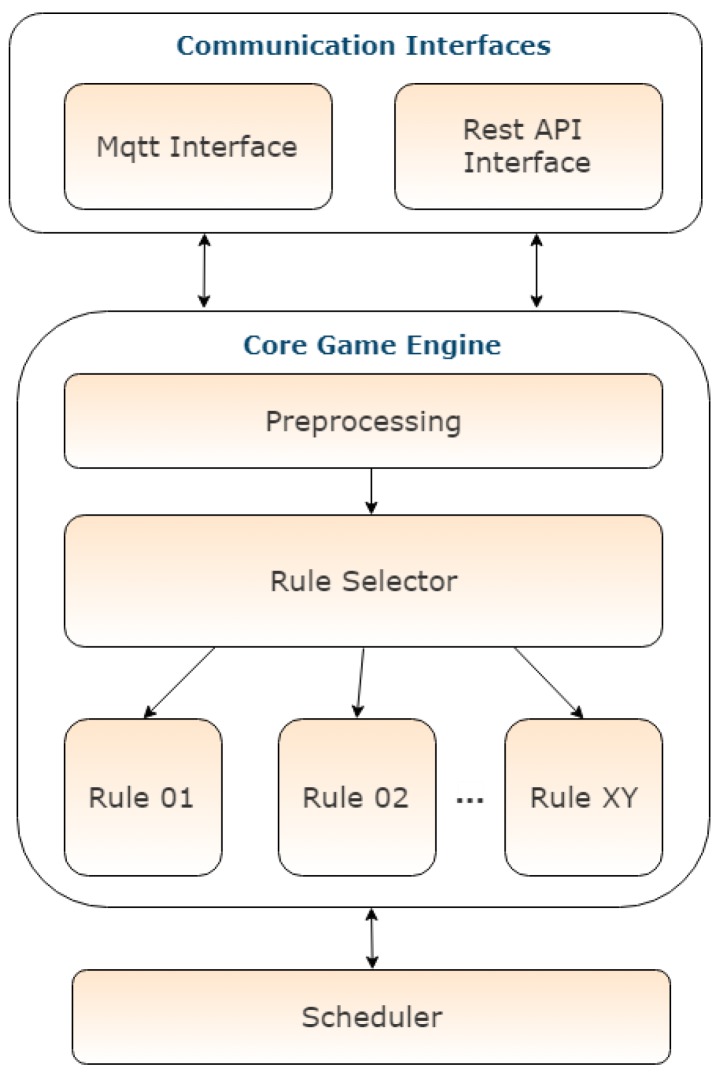
Game backend internal architecture.

The Game Backend, depicted in Figure 14, implements the game rules logic that is going to be used in order to decide the progress of a user in the game, update the user scores and leaderboard, and keep track of the currently accepted/available challenges of a user. It has been implemented in Java and interfaces with SiteWhere via MQTT and REST. All the communication between the software components happens through SiteWhere.

Whenever an event is sent to SiteWhere from a device (i.e., a measurement or an alert), the event is stored and forwarded to a predefined MQTT topic which is listened to by the other software components. The Game Backend listens to events sent to SiteWhere that describe the users’ behavior and its results (such as NFC swipes, user location updates and energy updates), processes the data and determines the user progress with respect to the challenges that have been accepted or schedules delayed, or recurrent, processing. The processing performed by the game backend is not necessarily tied with the specific time an alert has arrived. Separate logic can also be triggered or executed at a different time to check/update the game progress and provide updates to the other system components. Internally the Game Backend consists of three different subcomponents. The first one is responsible for interfacing with the rest of the system through MQTT and REST receiving input from other components and providing output regarding the current game state. The second sub-component is the core engine that implements the game logic. It includes a preprocessing module that handles the incoming events and accordingly selects the relevant rule in an asynchronous manner persisting the event state in disk. Each event (or alert) that arrives to the game backend contains a specific type and message identifying the nature of the event, which is then used by the preprocessor in order to deduce the required course of action (i.e., which rule to initialize). It also comprises the actual game rules, which are the main part of the game logic and model the game challenges or actions that are performed by the game backend. One such rule can be responsible for implementing a specific challenge according to which, for example, the user needs to turn off the lights and AC before leaving the office for the day. Another rule checks at the end of the week whether the weekly challenges (i.e., other rules), which have been undertaken by any user, have been accomplished or not. The third subcomponent is the scheduler. Its main use is to schedule delayed rules that should be executed at a future time or at specific time intervals (i.e., every day, every week, etc.). The input to the game backend comes from the mobile app, which sends alerts that correspond to user actions inside the building, from the energy analytics backend, which responds to requests for information regarding the energy outcome of the users’ actions, and also from SiteWhere where relevant information of the infrastructure is stored. This data is then processed by the game backend and the game state is updated accordingly (i.e., rules are validated, challenges are completed, the user profiles are updated with points earned, etc.). The updated game state is produced as output and it is sent to SiteWhere for storage in the database and also to the mobile app where it is displayed in the form of user notifications. New rules can be added as needed to incorporate new challenges. A separate submodule allows easily adding rules, thus ensuring the scalability and continuous enrichment of the game challenges. It organizes rules in a specific structure by inheriting from an abstract class Rule, which defines a common interface as well as implements common functionality.

**Figure 15:**
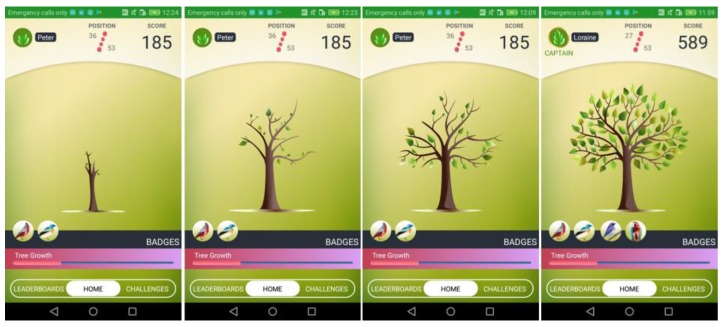
Screenshots of the mobile app.

The Mobile App (illustrated in Figure 15) is the end-user front-end and visualizes data and game challenges in a user friendly, appealing, modern and motivating interface to ensure continuous engagement. The app is designed for Android smartphones supporting API Layer 21 (Lollipop) and above, equipped with NFC and BLE capabilities. The gamified app visualizes information about energy behavior both at user and team level. Users are informed about their progress while their actions directly contributing to the energy impact can be traced. Achieving energy savings and accomplishing challenges results in accumulating scores. A visual emotional inceptive in the form of a living tree grows and prospers according to the users’ score, thus rendering the game also visually attractive and engaging. When a challenge is completed, the scores and the game progress for each user and their team are updated in real time between the backend and the mobile app. The game backend has been installed on the project server and connected with the MQTT broker, and through it, with the other software components. Furthermore, the design goal of the backend is to work on the background and send notifications via MQTT whenever there is a new update.

## 9. Component for Microgeneration Energy Forecasting

**Figure 16:**
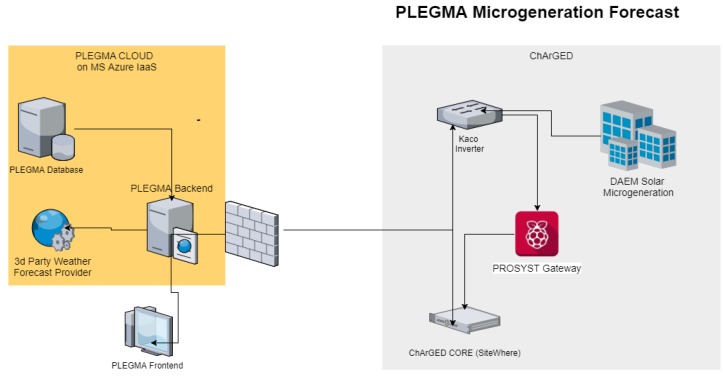
Solar PV prediction architecture.

This component utilizes a solar inverter (KACO new energy GmbH, Neckarsulm, Germany) with rich data communication capabilities (over Modbus TCP protocol) in order to monitor the generated electricity and assist the energy production forecasting mechanism which is based on daily weather forecasts. This forecasting is used for directing the game challenges towards optimizing energy use. The solar inverter is connected to the Sensor Gateway with the middleware/IoT integration software mBS SH. Through the device abstraction of the mBS SH the data are sent via MQTT to the ChArGED core platform and from there they are made accessible to all other system components. The Component for Microgeneration Energy Forecasting gets periodically updated (every day for five days ahead) on the specific location weather forecast and provides the hourly forecast of the expected energy. The solar forecast software is connected to third-party weather forecast provider APIs for obtaining the weather forecast data (https://www.yr.no/, https://www.wunderground.com/, https://exm.gr/, etc.). The overall architecture of the solar-microgeneration forecasting component is depicted in Figure 16. This forecasting will be utilized to design game challenges that propose to the players to shift energy-demanding tasks, e.g., print, charge mobile phones, to times that we expect high solar energy.

The pilot site in Athens has installed a PV microgeneration system with a peak power of 4.88 kWp, on the roof of the building, consisting of 25 solar panels, generators, a DC electrical panel and a KACO Powador TL3 inverter.

## 10. Process View

This section deals with the dynamic aspects of our platform. The system operation revolves around three main process flows, depicted in the following with UML sequence diagrams.

### 10.1. Collection of Data from the Building via Meters and Environmental Sensors Connected to the Sensor Gateway

**Figure 17:**
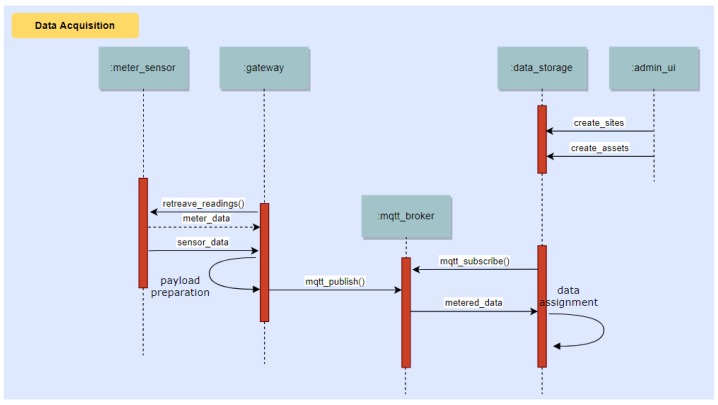
Sequence diagram showing the process of collecting data from building via meters and environmental sensors connected to the Sensor Gateway.

Metered data are communicated to the Data/Core Backend system via MQTT, and then assigned to Devices for use by other system components. Sites, devices and assets are created beforehand via the Administrative User Interface. This process is illustrated in Figure 17.

### 10.2. Claim of Energy Saving Achievements from the End-Users via the Mobile App

**Figure 18:**
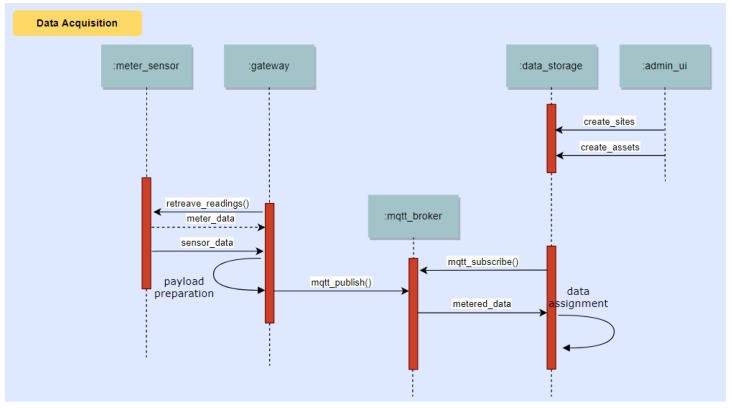
Sequence diagram showing the process of claiming energy saving achievements from the end-users via the Mobile App.

Energy measurements collected by the Sensor Gateway are sent to the Data/Core Backend system and from there forwarded to the Analytics backend for modelling of the energy use. When the mobile app reports user-triggered NFC and BLE events via MQTT or REST, such events are sent to the Data/Core Backend system and from there are also forwarded to the Analytics backend for modelling of the energy use as well as to the Game backend for accessing their effects on the game state. This process is illustrated in Figure 18.

### 10.3. Discovery of Energy Saving Opportunities to Assist the End Users in Saving Energy

**Figure 19:**
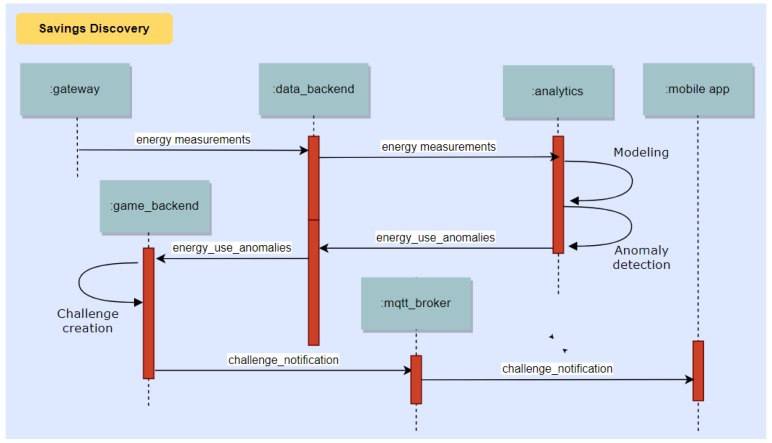
Sequence diagram showing the process of discovering energy saving opportunities and feeding such insights to the Game Engine.

Energy measurements collected by the Sensor Gateway are processed to create models of energy use. When new data are received, the Analytics backend compares them with the expected energy use at that time of the day and diagnoses deviations, which are in turn communicated to the Game backend. The Game backend uses this information to notify the end users if savings opportunities exist. This process is illustrated in Figure 19.

## 11. System Operation Example

**Figure 20:**
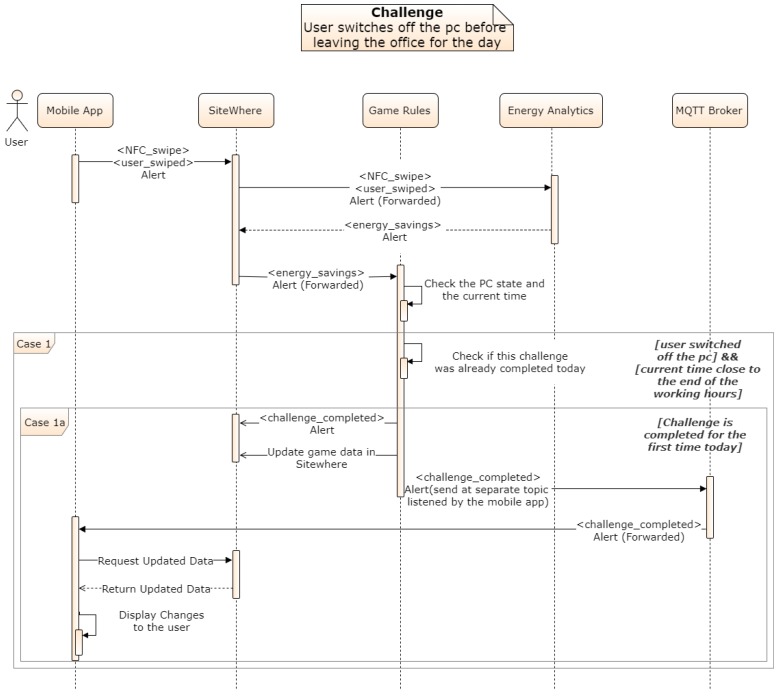
Sequence diagram of the game backend validation of user control actions.

This section presents an example of a rule that is handled by the Game and Energy Analytics Backend that checks if a user has closed the PC at the end of the day. Figure 20 depicts the sequence of events for the validation of a user control action, and the dissemination of this information to the involved components.

The following sequence of steps describes the sequence diagram of Figure 20:

1. User swipes an NFC Tag. An Alert Event is sent to the backend and connects the event with the user’s smartphone.
2. The Alert is forwarded to the game backend and the event is matched to the relevant device-NFC pair. Every device is linked to an NFC tag. The NFC id provided by the app is used to get the device_id from the metadata of the NFC. The swipe is also matched to the specific challenge and a unique challenge identifier is added to the alert.
3. Based on the device ID and NFC id association, the energy analytics backend retrieves the measurements, processes the data and produces an update at the backed with the calculated effect of the user’s action on the energy consumption. This is disseminated to the game mechanics.
4. The game backend subsequently then identifies a relevant specific challenge and compares the achievement with the challenge specification, for example a recognized change greater than a threshold (e.g., 15 W). If the requirements are met the challenge is considered completed and points are assigned to the user’s score.
5. The Game Backend finally disseminates the challenge completion request to a separate MQTT topic listened by the mobile app in order to communicate that there is an update available for the specific user. Then the mobile app parses these data and displays them in the front end.

## 12. Evaluation

### 12.1. Methodology

Our gamified system is being currently deployed at three pilot sites in three different countries, namely Luxembourg (Musée National d’Histoire et d’Art, referred to as MNHA), Spain (EcoUrbanBuilding, Institut Català d’Energia headquarters, Barcelona, referred to as ICAEN) and Greece (General Secretariat of the Municipality of Athens, referred to as DAEM).

The National Museum of History and Art (MNHA) is a public museum positioned at the heart of the old city of Luxembourg. MNHA comprises four buildings (the new building and three renovated old houses). The exhibition rooms’ space is 4300 m^2^ and there are 25 administrative offices. There are five floors and four underground levels where the museum’s archaeological collections are exhibited to the visitors. There are about 100 employees in MNHA that span various roles including administrative, IT, electricians and art-creative. Employees cannot alter the climate conditions in the exhibition area, which is suitable for art preservation. Heating is controlled per room by employees, while there is no A/C in the administrative offices. There are two lifts, while lighting in the exhibition area is centrally controlled. Regarding IT infrastructure in MNHA, there are PCs in the offices, shared printers per floor, some individual printers, fax machines and some shared plotters for printing graphics. Moreover, a kitchen for the staff includes a refrigerator, a microwave and coffee machines. Finally, there is one room where visitors interact with four touch screens to have a summary of Luxembourg history. Employees and visitors will participate in the trials of ChArGED.

ICAEN is the institute of energy of Catalonia. It is located at the 3rd floor of a 7-storey building covering an area of 1120 m^2^. At ICAEN, there are 49 employees in various roles (administration, energy efficiency and renewable energy systems, project management, communications, economics, regulator consultants and more). There are no visitors from the general public, excepting meetings arranged with ICAEN’s employees. There is a multitude of HVAC units in the building, however, temperature is centrally controlled within a range dictated by a legal directive, while heating is gas-powered. Moreover, there are two humidifiers in use and four spare ones for backup per floor. Lighting is manually controlled by employees. There is significant IT infrastructure at ICAEN comprising PCs, screens, shared printers and faxes. Three meeting rooms have big LCD screens (e.g., 60″). Two kitchen rooms are used by ICAEN employees that are equipped with boilers, microwave ovens, toasters, fridges, coffee machines, vending machines and a water cooler.

DAEM is the IT company of the municipality of Athens. The ChArGED application will be deployed at half of the 1st floor of a 6-storey building (DAEM’s HQ and offices)covering an area of 650 m^2^. The 55 employees of DAEM span across various functional units including IT, EU projects, sales, support, administration, management and technical. The electrical equipment in DAEM is that of a typical office building comprising HVAC units, lighting, PCs, laptops, printers, scanners, paper shredders, faxes, TVs, network switches and storage units. Moreover, the DAEM premises are equipped with kitchen appliances, such as refrigerators, water coolers, microwave ovens, coffee machines, etc. All electrical equipment is controlled by employees.

Following the principles of agile development, feedback from the end users will be continuous throughout the development and deployment processes. However, the system validation is organized in three phases each with different goals where specific feedback will be systematically elicited and recorded before moving to the subsequent phases. In the first validation phase (lasting 2 months), the objective is to carry out usability tests of individual prototype components, in order to obtain feedback from end users regarding their initial impression from the user interface of the Mobile App (not fully integrated), from the game mechanics and the artificial-life persona (potentially not fully integrated), as well as from each individual system component (not yet fully integrated). The objective of the second validation phase (lasting 2 months) is to carry out usability tests of the 1st integrated ChArGED system, in order to obtain feedback from end users regarding their impressions from using the mobile app (fully implemented and integrated with the rest of the system) for final improvements on the game features before the delivery of the final integrated system. In the third validation phase, i.e., the actual pilot trials (lasting 12 months), we aim to carry out usability and acceptance tests of the released ChArGED system. The feedback collected at this stage, will be used to improve the ChArGED system delivered at the end of the project. Ideally, 40 users from each pilot site will participate in the third validation phase.

In the pilot trials, our system will be evaluated with a number of Key Performance Indicators based on quantitative and qualitative metrics, including:

- *Energy Consumption Reduction*: It involves (a) measurement of energy use at site level in each pilot site for a period of 12 month prior to the introduction of ChArGED application, (b) creation of a baseline model of energy use for each site dependent on climate conditions (e.g., ambient temperature, humidity, etc.) and day of the week, (c) measurement of energy consumption with ChArGED system deployed and comparison with baseline energy consumption in the same conditions. We are particularly interested in the percentage reduction of energy wastage. This can be approximated based on the fraction of the energy impact of the accomplished challenges over the energy impact of the total challenges offered to the game participants. A satisfactory percentage reduction of wasted energy should be between 15% and 30% (according to the project goals), while we expect to achieve significantly higher percentage in reality.
- *Energy Awareness and Energy-Conservation Knowledge*: Utilizing carefully-designed survey instruments (i.e., questionnaires) before and after the ChArGED game intervention, we will collect user feedback regarding any enhancements on knowledge (i.e., know-how) for energy conservation and energy awareness (i.e., beliefs).
- *Behavioral Change*: Utilizing carefully-designed survey instruments (i.e., questionnaires), in this case as well, before and after pilot trials, we will compare self-reported energy-consumption behaviors of users with the ones they actually adhered to based on the game challenges they have undertaken and accomplished. We will measure the average number (e.g., daily/weekly/monthly) of identified energy conservation actions performed based on offered game challenges and the fraction of accomplished over offered game challenges over time. A satisfactory performance (according to the project goals) should be over 15–30% changes in actual behaviors against reported ones prior to the intervention, but we aim for an even higher percentage.
- *User Engagement*: This performance indicator aims to track the degree at which the users have included the use of the ChArGED solution in their daily routines. We will measure the average number of undertaken challenges over time across users, the average time per week day that the app has been used over time across users and the average number of times per day that the app has been accessed.
- *Scalability*: The scalability of our solution will be evaluated, among other metrics, in terms of throughput and latency of event/message processing versus the number of measurements and events produced over time.

### 12.2. Preliminary Results

**Figure 21:**
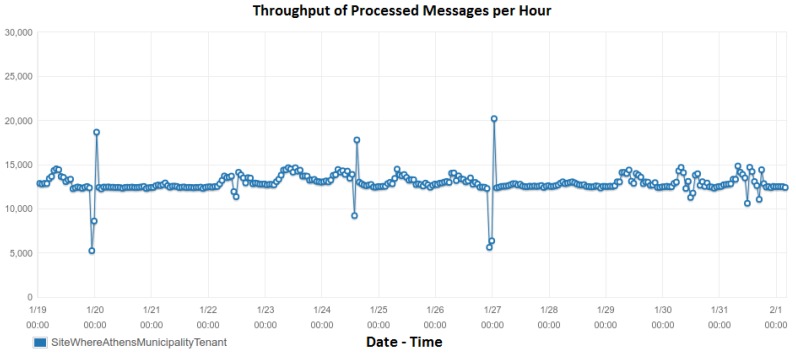
The number of messages (power measurements and sensor events from DAEM) received per hour by the Data/Core Backend over time and processed by the Energy Analytics Backend.

**Figure 22:**
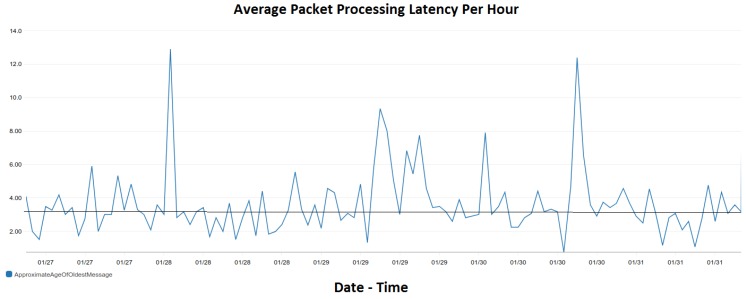
The average message processing latency per hour over time of the Energy Analytics Backend for the messages (power measurements and sensor events) received by DAEM.

The preliminary evaluation of our system focuses on scalability aspects. We employ the hardware installed at DAEM premises. The ChArGED system runs on a server with a quad-core Intel Xeon CPU X5550 running at 2.67 GHz and 16 GB of RAM. We receive power measurements from 54 points (circuit meters and smart plugs) every 15 s. The average packet size is 480 bytes. We also receive sensor events from BLE and NFC sensors, whenever they arise. The time it takes for SiteWhere (Data/Core Backend) to process a single sensor event or power measurement and send it to the Energy Analytics Backend is a few microseconds. We have found that up to 10,000 messages/second can be handled by the current system. If needed, the system can be horizontally scaled by moving resource-consuming software components to dedicated servers and further increase the throughput. Recall that the Energy Analytics Backend and Sitewhere are connected through Amazon SQS (Amazon.com, Inc., Seattle, WA, USA). All power measurements and sensor events are stored in SiteWhere and processed by the Energy Analytics Backend for creating baseline models and for user action validation, while NFC and BLE events are also used to trigger validation of challenges at the Game Backend. Figure 21 depicts the number of messages per hour over time received and processed by the Analytics Backend, as extracted by Amazon SQS. Observe that the Analytics Backend processes over 13,000 data packets per hour on the average, i.e., it processes all power measurements and sensor events produced. Note that this message rate is received only from DAEM and it is well below the capacity of our system (i.e., 10,000 messages/s). Also, the processing latency per packet is depicted in Figure 22. As it can be seen therein, the maximum average packet processing latency per hour is 13 s, while the average latency (illustrated with a solid straight line) is 3.35 s per packet, which is much lower than the 15-s period of power measurements.

## 13. Conclusions

This paper presents a gamified system framework that aims to change energy-consumption behavior and reduce wasted energy in public buildings. This framework exploits IoT enabled, low-cost devices to improve energy disaggregation mechanisms that provide energy use and-consequently-wastage at the device, area and end-user level. A gamified application has been developed to target these wastages and provide personalized real-time recommendations to each individual end user. This solution is being developed in the context of project ChArGED with iterative end-users’ engagement during analysis, design, development and validation in public buildings located in three different countries. Future work will involve the finalization of the integrated system towards its deployment in the three pilot sites and the complete design of the game mechanics that will be incorporated by the Mobile App, in order to be used on a daily basis in a 1-year trial/assessment period.
